# Malaria-anemia comorbidity and its determinants among pregnant women in high- and moderate-malaria-risk countries in Sub-Saharan Africa

**DOI:** 10.1186/s40249-025-01357-x

**Published:** 2025-08-13

**Authors:** Alebachew Ferede Zegeye, Mulugeta Wassie, Tadesse Tarik Tamir, Berhan Tekeba, Enyew Getaneh Mekonen, Gebreeyesus Abera Zeleke, Deresse Abebe Gebrehana

**Affiliations:** 1 Department of Medical Nursing, School of Nursing, College of Medicine and Health Sciences, University of Gondar, Gondar, Ethiopia; 2 Department of Nursing, School of Nursing, College of Medicine and Health Sciences, University of Gondar, Gondar, Ethiopia; 3 Department of Pediatrics and Child Health Nursing, School of Nursing, College of Medicine and Health Sciences, University of Gondar, Gondar, Ethiopia; 4 Department of Surgical Nursing, School of Nursing, College of Medicine and Health Sciences, University of Gondar, Gondar, Ethiopia; 5 Department of Internal Medicine, School of Medicine, College of Medicine and Health Sciences, University of Gondar, Gondar, Ethiopia

**Keywords:** Malaria, Anemia, Comorbidity, Pregnant women, Sub-Saharan Africa

## Abstract

**Background:**

Malaria and anemia remain a major public health problem in Sub-Saharan Africa, with pregnant women being particularly vulnerable to its adverse effects. Despite significant efforts to control malaria and anemia, the burden and adverse effects persist, especially in developing countries among pregnant women. Existing studies investigated malaria and anemia separately and identified individual-level factors as contributors to malaria or anemia, yet the influence of community-level factors remains underexplored. This study aimed to assess the malaria-anemia comorbidity and its determinants among pregnant women in high- and moderate-malaria-risk countries in Sub-Saharan Africa.

**Methods:**

Data from the Malaria Indicator Surveys (MIS) conducted between 2016 and 2022 across 17 Sub-Saharan African countries were used for analysis. The study included a total of 50,545 weighted samples. Multilevel logistic regression was used to assess individual and community-level factors associated with malaria-anemia comorbidity. Factors associated with malaria-anemia comorbidity were considered significant at *P*-values < 0.05. A model with the lowest deviance and highest log-likelihood ratio was selected as the best-fit model.

**Results:**

The pooled prevalence of malaria-anemia comorbidity among pregnant women was 39.00% (95% *CI* 29.00–49.00). No formal education (*OR* = 1.43, 95% *CI* 1.34–1.54), using untreated bed nets (*OR* = 1.23, 95% *CI* 1.16–1.30), poor wealth index (*OR* = 2.37, 95% *CI* 2.18–2.57), not using indoor residual spraying (*OR* = 2.15, 95% *CI* 1.87–2.48), households without a television (*OR* = 1.33, 95% *CI* 1.23–1.44), rural residence (*OR* = 2.73, 95% *CI* 2.54–2.93), and residing in West Sub-Saharan Africa (*OR* = 8.00, 95% *CI* 7.47–8.57), Central Sub-Saharan Africa (*OR* = 6.76, 95% *CI* 76.03–7.57), and South Sub-Saharan Africa (*OR* = 18.76, 95% *CI* 17.3–20.4) were determinants of malaria-anemia comorbidity.

**Conclusions:**

This study revealed high malaria-anemia comorbidity among pregnant women in high- and moderate-malaria-risk countries in sub-Saharan Africa, with both individual- and community-level factors as significant determinants. Health policies should prioritize targeted interventions for pregnant women, especially in rural areas, with an emphasis on increasing untreated bed net use, and region-specific strategies, particularly in West, Central, and South Sub-Saharan Africa, where malaria-anemia comorbidity burden is notably high.

## Background

Over the past few decades, malaria has been known as a serious public health problem, especially for pregnant women in malaria-endemic regions [1, 2]. Malaria is caused by five different *Plasmodium* species, such as *P. falciparum*, *P. vivax*, *P. malariae*, *P. knowlesi*, and *P. ovale* [3, 4]. Among *Plasmodium* species, *P. falciparum* and *P. vivax* account for the majority of malaria cases among pregnant women [5].

Anemia during pregnancy is the most severe and potentially fatal, which is defined by a hemoglobin concentration of less than 110 g/L [6, 7]. The World Health Organization (WHO) considers a population study to be of public health significance if the prevalence of anemia is ≥ 5%, and it is deemed to be a severe public health issue if the prevalence of anemia is ≥ 40% [8, 9]. In addition to being a major sign of inadequate nutrition and health, anemia reduces the blood’s ability to carry oxygen throughout the body [10] and is a major cause of maternal morbidity and mortality, accounting for over 115,000 maternal deaths annually in developing countries [11].

Malaria and anemia are significant public health concerns, particularly among pregnant women in Sub-Saharan Africa (SSA). In 2021, there were an estimated 40 million pregnancies across 38 malaria-endemic African countries, of which 13.3 million (32%) were exposed to malaria infection during pregnancy [12]. This exposure contributes to a substantial burden of maternal anemia, with an estimated 38% of pregnant women in the region affected [13]. The co-occurrence of malaria and anemia during pregnancy can lead to severe adverse outcomes, including increased risks of low birth weight, preterm birth, stillbirth, and neonatal and maternal mortality [14, 15].

Globally, an estimated 121.9 million pregnancies occurred in malaria transmission areas in 2020, with Sub-Saharan Africa accounting for approximately 46.1 million of these cases [16]. In Africa, severe maternal anemia, a frequent complication of malaria in pregnancy, contributes to about 10,000 maternal deaths annually [17]. In Sub-Saharan Africa, malaria significantly contributes to anemia during pregnancy, being responsible for up to 26% of severe anemia cases [18].

The WHO established recommendations for daily folic acid and iron supplementation to prevent iron deficiency-related anemia [19, 20], as well as widespread use of insecticide-treated nets (ITNs) and the administration of intermittent preventive treatment in pregnancy (IPTp) using sulfadoxine-pyrimethamine [21]. Coverage of these interventions has increased in recent years. Between 2017 and 2018, the proportion of pregnant women in Sub-Saharan Africa receiving three or more doses of IPTp rose from 22 to 31%, while ITN usage among pregnant women and children increased from 26% in 2010 to 61% in 2018 [22, 23]. Despite these efforts, malaria-anemia comorbidity during pregnancy remains a persistent public health challenge, with intervention coverage and effectiveness varying widely across regions.

The risk of malaria-anemia comorbidity among pregnant women is influenced by multiple demographic, health-related, and behavioral factors, including maternal educational status [24, 25], maternal age [26, 27], antenatal care (ANC) visits [27], parity [24], gravidity [28], gestational age [29], HIV status [13, 30], the use of ITNs [31, 32], and iron and folic acid supplementation [33].

Despite extensive research on malaria epidemiology, there is limited evidence on the malaria-anemia comorbidity specifically among pregnant women across SSA. Most studies have focused on general population-level malaria-anemia prevalence without identifying high-risk population groups such as pregnant women. Moreover, to the best of our knowledge, no study has systematically investigated the multilevel determinants of malaria-anemia comorbidity among pregnant women using Malaria Indicator Surveys (MIS) across SSA.

Therefore, this study aimed to fill this gap by examining malaria-anemia comorbidity and its determinants among pregnant women in high-and moderate-malaria-risk countries in Sub-Saharan Africa using malaria indicator surveys.

## Methods

### Study setting

This study included data from 17 countries in Sub-Saharan Africa: Burkina Faso, Cameroon, Ghana, Guinea, Kenya, Liberia, Madagascar, Mali, Malawi, Mozambique, Nigeria, Niger, Sierra Leone, Senegal, Togo, Tanzania, and Uganda. These countries were selected because they are the only countries with available MIS datasets between the given time period.

### Study design and period

A secondary analysis of MIS collected between 2016 and 2022 across 17 Sub-Saharan African countries was conducted. The MIS is a nationally representative survey program implemented by the respective national ministries of health in collaboration with international partners, primarily the Demographic and Health Surveys (DHS) Program, which is funded by the United States Agency for International Development (USAID), with technical assistance from an international coaching federation (ICF). The MIS is carried out approximately every 5 years, using pretested, validated, and structured tools. For this study, a 7-year dataset starting from 2016 was compiled by merging representative samples from each selected Sub-Saharan African country. Data extraction, cleaning, recoding, weighting, management of missing data, and analysis were conducted from February 04, 2025, to March 27, 2025.

### Data source and sampling

The data source for this study was the MIS collected between 2016 and 2022 across 17 Sub-Saharan African countries. The MIS provides valuable data on malaria-related indicators such as malaria cases, prevention practices, and access to diagnosis and treatment. The MIS used a stratified two-stage cluster design. In the first stage, enumeration areas (EAs) were selected based on probability proportional to size. In the second stage, a fixed number of households were systematically selected from each EA. To ensure the analysis accounted for the complex survey design, we used the “svyset” command in STATA, specifying the cluster identifier as the primary sampling unit (PSU) and the strata variable for stratification, while applying the sampling weights (v005) after normalizing them. Data were extracted from the household record (PR) file of the MIS, which contains individual-level records with key demographic, socioeconomic, and health-related variables. The dependent variables in this study are malaria and anemia status, which include malaria infection status measured through rapid diagnostic tests and anemia status based on hemoglobin level. The independent variables include both individual- and community-level variables. In our study, we accounted for the complex survey design by incorporating sampling weights, stratification, and clustering at both the MIS sample cluster and country levels in our multilevel logistic regression models. The study analyzed a weighted sample consisting of 50,545 pregnant women (Table 1).

**Table 1:** Sample size for malaria-anemia comorbidity and its determinants among pregnant women in high and moderate malaria risk countries in Sub-Saharan Africa, Malaria Indicator Surveys 2016–2022

Country	Year of survey	Unweighted sample (*n*)	Unweighted sample (%)	Weighted sample (*n*)	Weighted sample (%)
Burkina Faso	2017/18	2261	4.39	2233	4.42
Cameroon	2022	2558	4.97	2519	4.98
Ghana	2019	1584	3.08	1481	2.93
Guinea	2021	2058	4.00	2100	4.16
Kenya	2020	8070	15.68	7881	15.59
Liberia	2022	1521	2.96	1490	2.95
Madagascar	2016	3935	7.65	3795	7.51
Mali	2017	3283	6.38	3393	6.71
Malawi	2021	1286	2.50	1460	2.89
Mozambique	2018	2156	4.19	2253	4.46
Nigeria	2021	6162	11.98	6440	12.74
Niger	2021	2096	4.07	2285	4.52
Sierra Leone	2016	4254	8.27	4157	8.22
Senegal	2020/21	633	1.23	94	0.19
Togo	2017	1869	3.63	1675	3.31
Tanzania	2017	3178	6.18	3107	6.15
Uganda	2018/19	4553	8.85	4181	8.28
Total sample size		51,457	100.00	50,545	100.00

*n*: sample size

### Population

The source population of the study was all pregnant women aged 15–49 years in Sub-Saharan African countries during the survey period from 2016 to 2022. The study population was all pregnant women residing in the selected enumeration areas in included countries of Sub-Saharan Africa during the survey period for which recent MIS datasets are available.

### Inclusion and exclusion criteria

All pregnant women in the reproductive age (15–49 years) who were in the selected enumeration areas were included in the analysis. All pregnant women in the selected enumeration areas during the survey period with incomplete MIS survey data files were excluded from this study.

### Sampling procedure

**Fig. 1:**
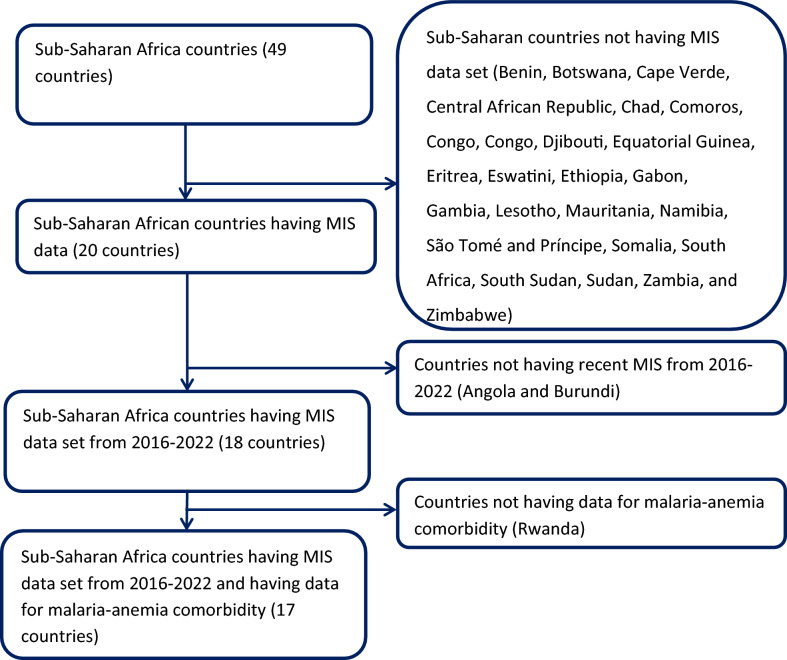
Sampling procedure for malaria-anemia comorbidity among pregnant women in high and moderate malaria risk countries in Sub-Saharan Africa

The MIS employs a stratified two-stage cluster sampling approach, first selecting EAs and then randomly sampling households within each EA. This study includes 17 Sub-Saharan African countries with MIS datasets from 2016 to 2024, systematically excluding 29 countries without Demographic and Health Surveys (DHS) data and one country lacking recent surveys from 2016 to 2024 (Fig. 1). The analysis has used the household record (PR) dataset to extract dependent and independent variables, and data were merged and processed in STATA (version 17, StataCorp LLC, College Station, TX, USA A key variable, v005, was used as a weighting variable.

### Study variables

Dependent variable: The outcome variables in this study were malaria infection and anemia. Malaria status was determined by the results of rapid diagnostic tests (RDT) or microscopy. The variable, malaria infection, was recoded into a binary variable where a value of 1 indicated a positive malaria infection (based on either a positive RDT or microscopy result), and a value of 0 represented a negative result (when both RDT and microscopy were negative) [34–36].

Anemia status was determined based on hemoglobin concentration, in accordance with World Health Organization (WHO) guidelines. Hemoglobin levels were categorized as follows: severe anemia (< 70 g/L), moderate anemia (70–99 g/L), mild anemia (100–109 g/L), and not anemic (≥ 110 g/L) [37]. For further analysis, anemia status was recoded into a binary variable: women with any level of anemia (severe, moderate, or mild) were coded as “yes,” while those classified as not anemic were coded as “no.”

To define malaria-anemia comorbidity, two binary variables were first created: (1) malaria infection, coded as 1 if the rapid diagnostic test or microscopy was positive and 0 otherwise; and (2) anemia, coded as 1 if the hemoglobin level was < 110 g/L and 0 if ≥ 110 g/L. These binary variables were then summed to create a composite score. A value of 2 indicated the presence of both malaria and anemia and was recoded as 1 (presence of comorbidity), while values of 0 or 1 were recoded as 0 (absence of comorbidity).

Independent variables: This study included two sources of independent variables due to the hierarchical nature of MIS data. The individual-level variables include factors such as maternal age, marital status, educational status, parity, ITN ownership, number of mosquito nets, ITN usage, type of bed net, use of IRS in the last year, wealth index, radio ownership, television ownership, type of main wall materials, type of main floor materials, and type of main roof materials. The community-level variables include media exposure, source of drinking water, type of place of residence, and country category.

### Operational definitions

Media exposure: It is determined by combining three survey variables: the frequency of reading newspapers or magazines, listening to the radio, and watching television. Individuals who engage with any of these media at least once a week are classified as having mass media exposure [38].

Wealth index: As defined by the malaria indicator surveys (MIS), it is a composite measure used to assess household socioeconomic status based on assets such as durable goods, housing characteristics, and access to services. It is constructed using Principal Component Analysis (PCA), which ranks households into wealth quintiles, from poorest to richest, based on the ownership of items like cars, televisions, and access to sanitation and clean water [39].

Water source: The source of drinking water is classified into improved and unimproved categories based on the WHO/UNICEF Joint Monitoring Programme (JMP) for Water Supply, Sanitation, and Hygiene. Improved water sources are those that are designed to protect against contamination, including piped water into dwellings, piped water to yards/plots, public fountains, boreholes with pumps, protected wells, protected springs, and rainwater. Conversely, unimproved water sources are those more susceptible to contamination, such as unprotected wells, unprotected springs, surface water (lakes, ponds, rivers, and irrigation channels), tanker trucks, and carts with small tanks [40].

Main roof materials are classified as unimproved and improved based on their durability and effectiveness in providing protection against weather elements. Unimproved roof materials include materials that are less durable and may provide insufficient protection, such as palm, bamboo, or mat. In contrast, improved roof materials are those that offer greater durability and better protection, indicating higher housing quality. These materials include wood planks, tarpaulin/plastic, zinc/metal, asbestos shingles, ceramic tiles, and concrete cement [41].

### Data management and processing

Data extraction, cleaning, recoding, and preparation were performed using STATA for analysis. Missing data were managed under the Missing at Random (MAR) assumption, which assumes that the probability of missingness depends on observed variables but not on the missing values themselves. To address missing data, we used the STATA command “drop if variable==.” warranting that only cases with complete data for key variables were included in the analysis. Then non-pregnant women’s data were removed from the dataset to focus exclusively on pregnant women. Following the cleaning process, the weighted proportions of the malaria and anemia status (the primary outcome variable) and the independent variables were computed in STATA.

In this study, we used survey design and clustering at the country and sample cluster levels throughout the entire analysis process. To make sure the estimates were representative of the target population, we used the svyset command in STATA to apply sample weights and account for clustering in the descriptive analysis. Stratification and clustering were taken into consideration in regression analysis by using survey-weighted models (with the svy prefix), which produced more accurate parameter estimates and accurate standard errors.

### Multilevel analysis

The data were extracted from the most recent MIS and processed using STATA. Before conducting any statistical analysis, the data were weighted using the women's weighting variable and stratum to ensure the survey's representativeness and account for the sampling design, thereby producing accurate standard errors and reliable statistical estimates. For survey-specific analyses, the weighting variable (hv005) was normalized. For pooled data, the women’s individual standard weight was denormalized by adjusting for the sampling fraction.

The standard logistic regression model assumptions, such as the independence of observations and equal variance, are violated due to the hierarchical structure of the MIS data. Specifically, individuals are nested within clusters, and participants within the same cluster are likely to share characteristics, differing from participants in other clusters. This clustering violates the assumptions of independence and equal variance, necessitating a more advanced approach. To address these challenges, hierarchical mixed-effects logistic regression was employed to identify factors associated with malaria-anemia comorbidity. Four models were used in the analysis: the null model, which included only the outcome variable to assess within-cluster variation in malaria-anemia comorbidity. Model I, which included only individual-level variables; Model II, which focused solely on community-level variables; and Model III, which incorporated both individual- and community-level variables. The null model provided a baseline for understanding cluster-level variation, while Models I and II separately examined the relationships between the outcome variable and individual- and community-level factors. The final model, Model III, simultaneously assessed the association between malaria-anemia comorbidity and factors at both individual- and community-level factors.

### Measure variation (random effects)

Random effects or measures of variation, including the likelihood ratio test (LR), intra-class correlation coefficient (ICC), and median odds ratio (m*OR*), were calculated to assess the variability in malaria-anemia comorbidity rates across clusters. By treating clusters as a random variable, the ICC was used to quantify the extent of heterogeneity in malaria-anemia comorbidity rates between clusters, representing the proportion of the total observed variation in malaria-anemia comorbidity attributable to differences between clusters. The ICC was computed using the formula ICC=$\frac{VC}{VC+3.29}\times 100\%$ [42]. The MOR quantifies the variation or heterogeneity in malaria-anemia comorbidity rates between clusters on the odds ratio scale. It represents the median value of the odds ratio when comparing a cluster with a higher likelihood of malaria-anemia comorbidity rates to a cluster with a lower likelihood, based on randomly selected individuals from each cluster: MOR = *e*^0.95√VC^ [43].

Additionally, the Proportional Change in Variance (PCV) indicates the variation in malaria-anemia comorbidity rates explained by the determinants, calculated as follows: PCV =$\frac{Vnull-Vc}{Vnull}\times 100\%$ [44], where Vnull represents the variance of the null model, and V_C_ is the cluster-level variance. Fixed effects were applied to estimate the association between malaria-anemia comorbidity and individual-and community-level independent variables. The strength of these associations was assessed using adjusted odds ratios (a*OR*) and 95% confidence intervals, with statistical significance determined at a *P*-value of < 0.05. Due to the nested structure of the model, deviance (calculated as − 2*log-likelihood ratio) was used to compare models, and the model with the lowest deviance and highest log-likelihood ratio was chosen as the best fit.

## Results

### Socio-demographic, economic, and health service related characteristics of pregnant women in Sub-Saharan Africa, MIS 2016–2022

In this study, a total weighted sample of 50,545 pregnant women was included. More than half of women (58.15%) had no formal education. More than two-thirds (78.38%) of women had ITN ownership, but nearly one-third (32.29%) of them used it, and about three-fourths (75.37%) of women were living in rural areas of Sub-Saharan Africa (Table 2).

**Table 2:** Socio-demographic, economic, and health service related characteristics of pregnant women in Sub-Saharan Africa, Malaria Indicator Surveys 2016 − 2022

Variables	Frequency (*n*)	Percent (%)
Individual-level factors		
Maternal age		
15–19	216	0.42
20–34	13,811	26.84
35–49	37,428	72.74
Educational status		
No education	29,923	58.15
Primary	12,220	23.75
Secondary and above	9312	18.10
Parity		
None	444	0.88
1–2	41,471	82.05
≥ 3	8630	17.07
ITN ownership		
No	10,927	21.62
Yes	39,618	78.38
Number of mosquito net		
None	19,704	38.98
One	14,837	29.36
Two and above	16,004	31.66
ITN used		
No	34,225	67.71
Yes	16,320	32.29
Type of bed net		
Untreated	23,410	46.31
Treated	27,135	53.69
Use of IRS in the last 1 year		
No	49,634	96.46
Yes	1,821	3.54
Wealth index		
Poor	23,632	46.75
Middle	10,431	20.64
Rich	16,482	32.61
Household has radio		
No	24,901	49.27
Yes	25,644	50.73
Household has television		
No	36,120	71.46
Yes	14,425	28.54
Main roof material		
Unimproved	28,355	56.10
Improved	22,190	43.90
Main floor material		
Unimproved	29,124	57.62
Improved	21,421	42.38
Main wall material		
Unimproved	32,582	64.46
Improved	17,963	35.54
Community-level factors		
Media exposure		
No	21,140	41.82
Yes	29,405	58.18
Source of drinking water		
Unimproved	25,064	49.59
Improved	25,481	50.41
Type of place of residence		
Urban	12,449	24.63
Rural	38,096	75.37
Country		
Burkina Faso	2233	4.42
Cameroon	2518	4.98
Ghana	1481	2.93
Guinea	2100	4.16
Kenya	7881	15.59
Liberia	1490	2.95
Madagascar	3795	7.51
Mali	3393	6.71
Malawi	1460	2.89
Mozambique	2253	4.46
Nigeria	6440	12.74
Niger	2285	4.52
Sierra Leone	4157	8.22
Senegal	94	0.19
Togo	1675	3.31
Tanzania	3107	6.15
Uganda	4183	8.28

*ITN* Insecticide-treated net, *IRS* indoor residual spraying

### Malaria-anemia comorbidity among pregnant women in high and moderate malaria risk countries in Sub-Saharan Africa

**Fig. 2:**
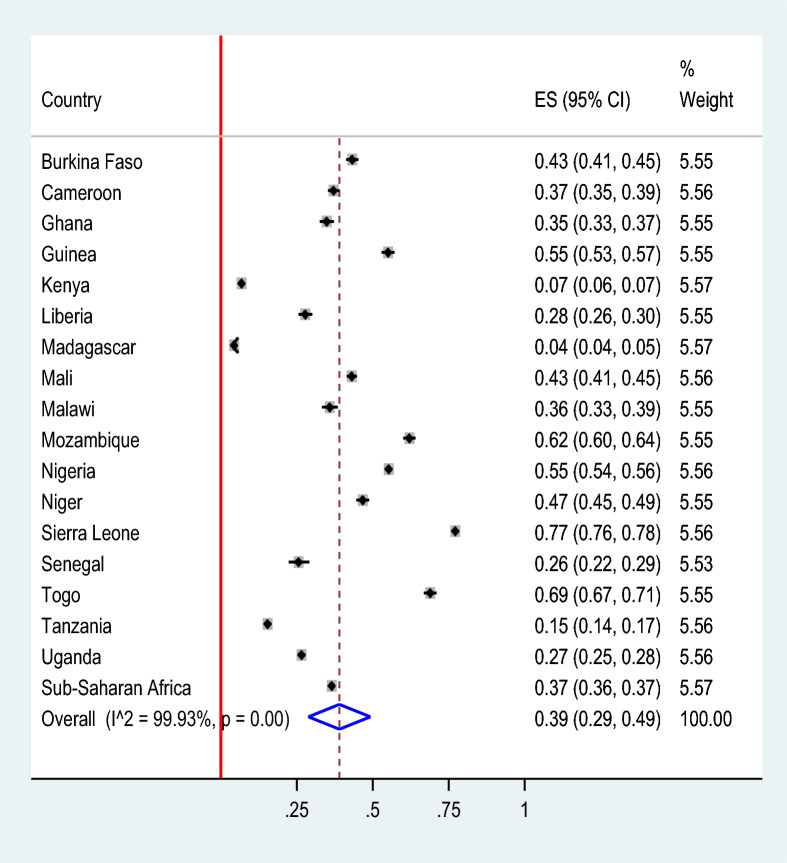
The pooled prevalence of malaria-anemia comorbidity among pregnant women in high and moderate malaria risk countries in Sub-Saharan Africa: Malaria Indicator Surveys 2016 − 2022

**Fig. 3:**
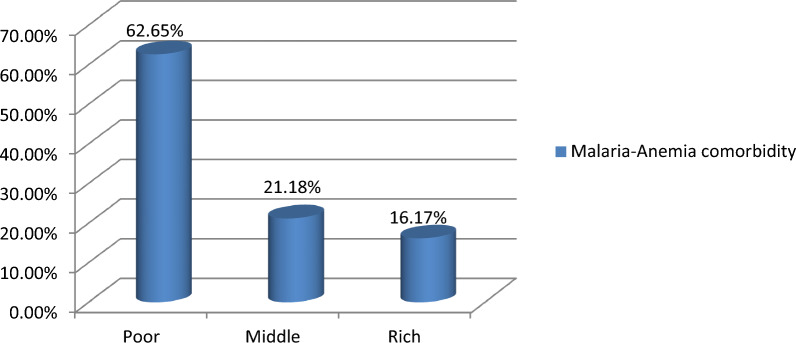
Prevalence of malaria-anemia comorbidity in relation to wealth quantiles among pregnant women in Sub-Saharan Africa: Malaria Indicator Survey 2016–2022

The pooled prevalence of malaria-anemia comorbidity among pregnant women in high- and moderate-malaria-risk countries in Sub-Saharan Africa was 39.00% (95% *CI* 29.00–49.00). Significant regional differences of malaria-anemia comorbidity were observed, with the lowest prevalence in Madagascar (4%) and the highest in Sierra Leone (77%) (Fig. 2). The prevalence of malaria-anemia comorbidity among pregnant women varied by wealth quintile, with rates of 62.65% among those in the poor quintile, 21.18% in the middle quintile, and 16.17% in the rich quintile (Fig. 3).

### Parameters of heterogeneity and model comparison

The null model, with a variance of 2.938897, revealed significant variations in malaria-anemia comorbidity among pregnant women in high- and moderate-malaria-risk countries in Sub-Saharan Africa. It indicated that approximately 47.18% of the overall variation in malaria-anemia comorbidity at the cluster level is attributable to community-level factors.

Additionally, when comparing an individual from a higher-risk cluster with one from a lower-risk cluster, the null model showed the highest median odds ratio (MOR) value of 5.10, meaning that individuals in higher-risk clusters have 5.10 times greater odds of contracting malaria-anemia comorbidity.

Model I’s intraclass correlation (ICC) explained 46.66% of the variation in malaria-anemia comorbidity rates between communities, while Model II ICC accounted for 24.22% of the variation. The final model, Model III, explained 22.62% of the variation in malaria-anemia comorbidity likelihood, considering both individual and community-level factors. Model III was the best-fitting model, as it had the lowest deviance (50,086.696) and the highest log-likelihood ratio (− 25,043.348) (Table 3).

**Table 3:** Parameters of heterogeneity and model comparison analysis for malaria-anemia comorbidity among pregnant women in high and moderate malaria risk countries in Sub-Saharan Africa

Parameter	Null model	Model I	Model II	Model III
Variance constant	2.938897	2.877478	1.05164	0.9617528
Interacluster correlation	47.18%	46.66%	24.22%	22.62%
Median odds ratio	5.10	5.01	2.65	2.54
Proportional change in variance	Reference	2.09%	64.22%	67.28%
Model comparison (model fitness)				
Logliklihood ratio	− 30,667.298	− 28,494.419	− 25,736.243	− 25,043.348
Deviance	61,334.596	56,988.838	51,472.486	50,086.696

### Factors associated with malaria-anemia comorbidity among pregnant women in high and moderate malaria risk countries in Sub-Saharan Africa

In a multilevel logistic regression analysis, maternal educational status, untreated bed net utilization, poor wealth index, non-utilization of indoor residual spraying (IRS), household television ownership, place of residence, and residing in western, central, and southern Sub-Saharan Africa were significantly associated with malaria-anemia comorbidity among pregnant women at a* P*-value of < 0.05.

The odds of malaria-anemia comorbidity are higher among pregnant women with no education (*OR* = 1.43, 95% *CI* 1.34–1.54) compared to those with secondary or higher education. The odds of malaria-anemia comorbidity were higher among women who used untreated bed nets (*OR* = 1.23, 95% *CI* 1.16–1.30) compared to women who used insecticide-treated bed nets. The odds of malaria-anemia comorbidity were higher for women with a poor wealth index (*OR* = 2.37, 95% *CI* 2.18–2.57) compared to those with a rich wealth index.

The odds of malaria-anemia comorbidity are higher among pregnant women who did not use indoor residual spraying (IRS) in the last year compared to those who did (*OR* = 2.15, 95% *CI* 1.87–2.48). Women in households without a television had higher odds of malaria-anemia comorbidity (*OR* = 1.33, 95% *CI* 1.23–1.44) compared to pregnant women who had one. The odds of malaria-anemia comorbidity were also higher for women living in rural areas (*OR* = 2.73, 95% *CI* 2.54–2.93) compared to those in urban areas. Regionally, the odds of malaria-anemia comorbidity were higher in West Sub-Saharan Africa (*OR* = 8.00, 95% *CI* 7.47–8.57), Central Sub-Saharan Africa (*OR* = 6.76, 95% *CI* 6.03–7.57), and South Sub-Saharan Africa (*OR* = 18.76, 95% *CI* 17.3–20.4) compared to pregnant women who reside in the East Sub-Saharan Africa region (Table 4).

**Table 4:** Determinants of malaria-anemia comorbidity among pregnant women in high and moderate malaria risk countries in Sub-Saharan Africa: Malaria Indicator Surveys 2016 − 2022

Individual level variables	Model I a*OR* (95% *CI*)	Model II a*OR* (95% *CI*)	Model III a*OR* (95% *CI*)	*P*-value
Maternal age		–		
15–19	0.80 (0.59–1.09)		0.79 (0.56–1.11)	0.175
20–34	1		1	
35–49	0.87 (0.65–1.18)		0.79 (0.56–1.11)	0.173
Educational status		–		
No education	1.60 (1.50–1.71)		**1.43 (1.34**–**1.54)**	0.000
Primary	1.02 (0.95–1.10)		1.38 (0.27–1.49)	0.070
Secondary and above	1		1	
Parity		–		
None	1		1	
1–2	1.39 (1.09–1.78)		1.08 (0.83–1.41)	0.551
≥ 3	1.97(1.53–2.52)		1.35 (1.04–1.76)	0.027
Bed net ownership		–		
No	1.23(1.15–1.31)		1.06 (0.99–1.14)	0.082
Yes	1		1	
Bed net utilized		–		
No	0.56 (0.53–1.59)		0.72 (0.68–1.76)	0.900
Yes	1		1	
Type of bed net		–		
Untreated	1.30 (1.23–1.38)		**1.23 (1.16**–**1.30)**	0.000
Treated	1		1	
Use of IRS in the last 1 year		–		
No	3.28 (2.89–3.72)		**2.15 (1.87**–**2.48)**	0.000
Yes	1		1	
Wealth index		–		
Poor	3.47 (3.22–3.73)		**2.37 (2.18**–**2.57)**	0.000
Middle	2.33 (2.17–2.49)		1.76 (0.63–1.90)	0.080
Rich	1		1	
Household has radio		–		
No	1.01(0.97–1.06)		0.98 (0.88–1.09)	0.741
Yes	1		1	
Main wall material		–		
Unimproved	0.94 (0.89–0.99)		1.02 (0.97–1.08)	0.453
Improved	1		1	
Main floor material		–		
Unimproved	0.83 (0.78–0.87)		1.00 (0.94–1.06)	0.953
Improved	1		1	
Community-level variables				
Community media exposure				
No	–	1.50 (1.44–1.57)	1.08 (0.95–1.21)	0.233
Yes		1	1	
Source of drinking water	–			
Unimproved		1.06 (1.00–1.11)	0.98 (0.93–1.02)	0.312
Improved		1	1	
Type of place of residence	–			
Urban		1	1	
Rural		4.53 (4.25–4.83)	**2.73 (2.54**–**2.93)**	0.000
Country category	–			
East SSA		1	1	
West SSA		7.75 (7.27–8.26)	**8.00 (7.47**–**8.57)**	0.000
Central SSA		6.20 (5.57–6.90)	**6.76 (6.03**–**7.57)**	0.000
South SSA		20.23 (18.7–21.9)	**18.76 (17.3**–**20.4)**	0.000

a*OR* adjusted odds ratio, *CI* confidence interval, *SSA* Sub-Saharan Africa, *OR* Odds Ratio, – Not applicable

NB: bold numbers indicate statistical significance

Multicollinearity was assessed using variance inflation factors (VIF), and the mean VIF value was found to be 2.00, indicating no significant multicollinearity among the explanatory variables.

## Discussion

In this study, the pooled prevalence of malaria-anemia comorbidity among pregnant women in high-and moderate-malaria-risk countries in Sub-Saharan Africa was 39.00% (95% *CI* 29.00–49.00). This finding was found to be higher compared to the previous studies conducted in Ghana, 10.5% [45, 46], Uganda, 20.1% [47], and India, 29.3% [48]. The discrepancies in malaria-anemia comorbidity burden among pregnant women across regions could be attributed to several contextual and methodological factors. Firstly, the variation in malaria transmission intensity and seasonality plays a major role; countries such as Ghana and Uganda have adopted aggressive malaria control programs, including widespread insecticide-treated bed net (ITN) distribution, intermittent preventive treatment in pregnancy (IPTp), and indoor residual spraying, which may have reduced malaria incidence and thus its hematologic consequences [49, 50]. Secondly, the differences in diagnostic tools and study designs can affect prevalence estimates. For instance, studies using rapid diagnostic tests (RDTs) or microscopy may underreport low-density parasitaemia compared to molecular techniques like PCR [51]. Thirdly, disparities in antenatal care coverage, nutritional status of women, prevalence of other infections (such as HIV or helminths), and health system effectiveness across settings also contribute to the observed variation. Additionally, the lower prevalence in India compared to this study finding could be the differences in host immunity, malaria species, and regional socioeconomic factors.

The prevalence of malaria-anemia comorbidity in this study was lower than in the studies conducted in Burkina Faso, 54.4% [52]; Cameroon, 40% [46]; Nigeria, 94.1% [53]; and Sub-Saharan Africa, 56% [13]. This study used nationally representative Malaria Indicator Survey (MIS) data, which incorporates both high-and low-transmission areas, potentially reducing the overall prevalence compared to studies focusing solely on high-burden regions. In addition, differences in diagnostic methods may have influenced the results; while MIS data often rely on rapid diagnostic tests (RDTs) and hemoglobin levels from finger-prick samples, facility-based studies may use more sensitive diagnostic tools such as microscopy or PCR, which could detect more cases [54]. Moreover, temporal differences may play a role, as some of the earlier studies may have been conducted during periods of higher malaria transmission or before intensified malaria control efforts were fully implemented [55].

In a multivariable multilevel logistic regression analysis, maternal educational status, untreated bed net utilization, poor wealth index, non-utilization of indoor residual spraying (IRS), household television ownership, place of residence, and residing in western, central, and southern Sub-Saharan Africa were significantly associated with malaria-anemia comorbidity among pregnant women at a *P*-value of < 0.05.

The odds of malaria-anemia comorbidity are higher among pregnant women with no education (*OR* = 1.43, 95% *CI* 1.34–1.54) compared to those with secondary or higher education. This finding is supported by the studies conducted in Nigeria [56, 57], Myanmar [58], India [59], and the USA [13]. This is due to findings that lower educational attainment is often associated with limited health literacy, which affects women’s knowledge and practices related to malaria prevention and maternal health, such as the use of insecticide-treated nets, timely antenatal care visits, and adherence to iron supplementation. Inadequate education also correlates with poor socioeconomic conditions, which further restrict access to healthcare services, nutritious food, and preventive interventions, increasing vulnerability to both malaria and anemia [60, 61].

The odds of malaria-anemia comorbidity were higher among women who used untreated bed nets (*OR* = 1.23, 95% *CI* 1.16–1.30) compared to women who used insecticide-treated bed nets. This result aligns with previous studies in Kenya [62], Ghana [63, 64], and Malawi [65]. The possible rationale for the association between higher malaria-anemia comorbidity and non-use of bed nets among pregnant women could be attributed to the reduced protection against mosquito bites and subsequent Plasmodium infection, which is a leading cause of anemia in endemic regions. ITNs not only provide a physical barrier but also kill or repel mosquitoes through insecticidal action, thereby significantly lowering malaria transmission risk [66]. In contrast, untreated nets lack this dual function, offering limited protection and increasing the likelihood of repeated infections that can result in hemolysis and iron depletion, key pathways leading to anemia during pregnancy [67].

The odds of malaria-anemia comorbidity were higher for women with a poor wealth quantile (*OR* = 2.37, 95% *CI* 2.18–2.57) compared to those with a rich wealth quantile. This finding is in line with the previous studies conducted in Ghana [8], Nigeria [68], and Tanzania [69]. The possible justification could be that women from poorer households often face limited access to preventive and curative health services, including insecticide-treated bed nets, prompt malaria treatment, and iron supplementation, which increases their vulnerability to both malaria and anemia. Additionally, poor nutritional status, which is more prevalent among economically disadvantaged populations, exacerbates susceptibility to anemia and weakens immune response to malaria infection. Socioeconomic disparities also influence health-seeking behavior and healthcare accessibility, further compounding the risk of comorbidity among low-wealth individuals [70–72].

The odds of malaria-anemia comorbidity are higher among pregnant women who did not use indoor residual spraying (IRS) in the last year compared to those who did (*OR* = 2.15, 95% *CI* 1.87–2.48). This finding is supported by the studies conducted in Burkina Faso [73], Guinea [74], and India [75]. The potential explanation could be that IRS helps reduce the vector population, thereby decreasing malaria incidence, which in turn lowers the likelihood of developing anemia due to the parasitic infection [76]. Women in households without a television had higher odds of malaria-anemia comorbidity (*OR* = 1.33, 95% *CI* 1.23–1.44) compared to pregnant women who had one. The finding is in line with the studies done in Uganda [77], Canada [78], and Latin America [79]. This association could be that television serves as a vital source of public health education, providing messages about malaria prevention strategies, such as the importance of using insecticide-treated bed nets, accessing antenatal care, and recognizing early symptoms. Pregnant women in households with televisions are more likely to be exposed to these educational campaigns, increasing their awareness and adoption of protective measures [80]. Conversely, households without television may lack access to such critical health information, leading to lower knowledge levels and fewer preventive actions [81].

The odds of malaria-anemia comorbidity were also higher for women living in rural areas (*OR* = 2.73, 95% *CI* 2.54–2.93) compared to those in urban areas. This finding is in line with other studies in Guinea [82], India [83], France [84], and Brazil [85]. The higher odds of malaria-anemia comorbidity among pregnant women living in rural areas compared to urban areas can be attributed to several interrelated factors, including limited access to healthcare services, lower socioeconomic status, and poorer infrastructure in rural settings. Rural areas often face challenges such as inadequate health facilities, lower availability of malaria prevention tools like insecticide-treated nets, and limited access to antenatal care, which contribute to the higher risk of both malaria and anemia [86, 87].

Regionally, the odds of malaria-anemia comorbidity were higher in West Sub-Saharan Africa (*OR* = 8.00, 95% *CI* 7.47–8.57), Central Sub-Saharan Africa (*OR* = 6.76, 95% *CI* 76.03–7.57), and South Sub-Saharan Africa (*OR* = 18.76, 95% *CI* 17.3–20.4) compared to pregnant women who reside in the East Sub-Saharan Africa region. The justification for these regional differences could be attributed to several factors, including variations in the prevalence of malaria, access to healthcare, and differences in nutritional status among pregnant women. Malaria transmission is notably higher in certain regions of Sub-Saharan Africa, particularly in West, Central, and South Sub-Saharan Africa, where environmental factors such as higher temperatures, stagnant water, and greater vector populations contribute to more frequent malaria outbreaks [88].

Furthermore, maternal anemia in these regions is exacerbated by poor dietary intake, limited access to iron supplementation, and other micronutrient deficiencies, which are more prevalent in certain areas compared to the East [89]. The healthcare infrastructure in some of these regions may also be less strong, leading to inadequate preventive measures such as intermittent preventive treatment for malaria (IPTp) during pregnancy [90]. Moreover, differences in regional malaria control strategies, such as insecticide-treated nets (ITNs) distribution and the use of artemisinin-based combination therapies, might account for the higher comorbidity observed in these regions [91]. In contrast, the East Sub-Saharan Africa region has had relatively better access to malaria prevention and treatment interventions, possibly reducing the burden of malaria and its associated anemia in pregnant women [92].

The strength of this study lies in the use of recent large-sample national Malaria Indicator Surveys (MIS) to assess the malaria-anemia comorbidity and its determinants among pregnant women in high-and moderate-malaria-risk countries in Sub-Saharan Africa. The application of mixed multilevel logistic regression, which enabled the identification of two-level factors that standard logistic regression, could not do. Despite the strengths of using large, nationally representative Malaria Indicator Survey (MIS) data and robust multilevel modeling, several limitations must be acknowledged. First, the cross-sectional nature of the MIS data restricts our ability to establish causal relationships between exposure variables and malaria-anemia comorbidity. Associations observed in this study should therefore be interpreted as correlational rather than causal. Second, although multiple relevant covariates were included in our models, the potential for residual confounding remains. Important factors such as maternal nutritional status, presence of coinfections, micronutrient supplementation, and environmental variables such as temperature, rainfall, and proximity to mosquito breeding sites were not available in the dataset and could influence the observed associations.

## Conclusions

The malaria-anemia comorbidity among pregnant women in high- and moderate-malaria-risk countries in Sub-Saharan Africa was found to be high compared to the WHO report, where malaria-anemia as a public health problem is defined as having a prevalence of ≥ 5% as being of public health significance and ≥ 40% as indicating a severe public health concern. With both individual and community-level factors significantly associated with malaria-anemia comorbidity. Based on these findings, we recommended that health policies in Sub-Saharan African countries prioritize targeted interventions for pregnant women, particularly in rural areas, where access to healthcare and prevention measures is limited. Strategies should focus on improving the use of bed nets and indoor residual spraying (IRS), enhancing education, and implementing region-specific measures, such as in West Sub-Saharan Africa, Central Sub-Saharan Africa, and South Sub-Saharan Africa countries.

## Data Availability

Third party data was obtained for this study from the DHS Program (https://dhsprogram.com/). Data may be requested from The DHS Program after creating an account and submitting a concept note. More access information can be found on The DHS Program website (https://dhsprogram.com/data/Access-Instructions.cfm). The data set is openly available upon permission from the MEASURE DHS website (https://www.dhsprogram.com/data/available-datasets.cfm). The authors confirm that interested researchers would be able to access these data in the same manner as the authors. The authors also confirm that they had no special access privileges that others would not have.

## Abbreviations

a*OR*: Adjusted odds ratio
ANC: Antenatal care
*CI*: Confidence interval
DHS: Demographic and Health Surveys
EA: Enumeration AREA
HIV: Human immunodeficiency virus
ICC: Intraclass correlation coefficient
ICF: International Coaching Federation
IPTp: Intermittent Preventive Treatment in Pregnancy
IRS: Indoor residual spraying
ITN: Insecticide-treated net
JMP: Joint Monitoring Programme (WHO/UNICEF)
LR: Likelihood ratio
MAR: Missing at random
MIS: Malaria indicator survey
m*OR*: Median odds ratio
*OR*: Odds ratio
PCA: Principal component analysis
PCR: Polymerase chain reaction
PCV: Proportional Change in Variance
PSU: Primary sampling unit
RDT: Rapid diagnostic test
SSA: Sub-Saharan Africa
USAID: United States Agency for International Development
VIF: Variance inflation factor
WHO: World Health Organization
